# Elderly Diabetic Patient with Surgical Site Mucormycosis Extending to Bowel

**DOI:** 10.4103/0974-777X.62877

**Published:** 2010

**Authors:** Atul K Patel, Himanshu J Vora, Ketan K Patel, Bhavin Patel

**Affiliations:** 1 *Infectious Diseases Clinic, “Vedanta” Institute of Medical Sciences, Ahmedabad, India*; 2 *Sterling Hospital, Ahmedabad, India*

**Keywords:** Gastrointestinal mucormycosis, Intra-abdominal infection, Mucormycosis, Surgical site infection

## Abstract

Mucormycosis is rare in clinical practice. Most infections are acquired by inhalation; other portals of entry are traumatic implantation and ingestion in immunocompromised host. Mucormycosis is life threatening infection in immunocompromised host with variable moratlity ranging from 15-81% depending upon site of infection. General treatment principles include early diagnosis, correction of underlying immunosuppression and metabolic disturbances, adequate surgical debridement along with amphotericin therapy. We describe surgical site mucormycosis extended to involve large bowel in elderly diabetic patient.

## INTRODUCTION

Invasive infections caused by the Zygomycetes are well known to clinicians and now increasing in numbers. Within the class Zygomycetes, the order Mucorales contains the genera *Rhizopus, Mucor,* and *Rhizomucor,* which cause most cases of human infection. Mucormycosis is a life-threatening infection caused by fungi of the order Mucorales. These fungi are found in soil, in decaying vegetation, in manure, and on a variety of foodstuffs, including bread, fruits, and seeds.[1–3] Most infections are acquired by inhalation; traumatic implantation and ingestion also are portals of entry.[1,2,4] Risk factors for development of infection with the zygomycetes include poorly controlled diabetes mellitus, hematologic malignancies (especially with neutropenia), receipt of a solid-organ or hematopoietic stem cell transplant (HSCT), deferoxamine therapy for iron or aluminum overload states, burn wounds and corticosteroid therapy.[5,6] The incidence of mucormycosis is increasing due to increase in hematopoietic stem cell transplant recipients and patients with hematological malignancies. Mucormycosis is usually acute and progressive, with mortality rates in the range of 70%–100%. Gastrointestinal (GI) mucormycosis is rare and fatal disease, mostly described in patients with malnutrition, infants with low birth weight, patients receiving peritoneal dialysis, abdominal trauma and patients with solid organ transplants.[6] The stomach, colon and ileum are the most commonly involved sites. As GI involvement with this infection is acute and rapidly fatal, it is often diagnosed postmortem.[6] We describe a case of post-surgical intestinal and surgical wound mucormycosis in patient with well-controlled diabetes mellitus.

## CASE

**Figure 1:**
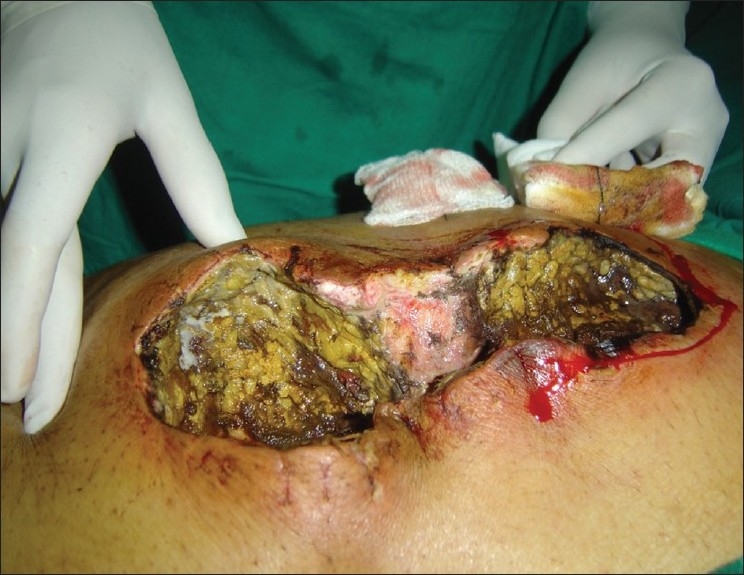
Intra operative picture showed black margins with surrounding erythema with extensive slough and white colony over bowels

**Figure 2:**
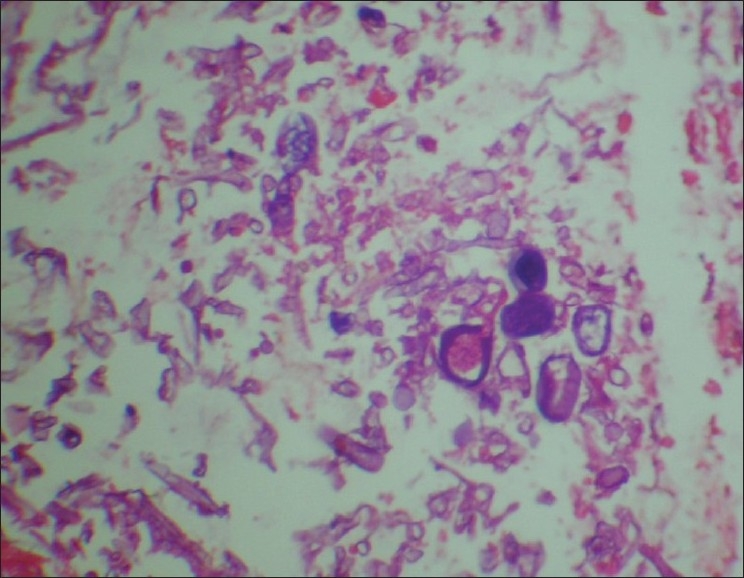
Microscopic examination showed broad, aseptate, right angle branching, ribbon like hyphae

A 75-year-old male was referred with history of abdominal surgery for ischemic bowels following mesenteric arterial insufficiency before 11 days. Patient was hypertensive and diabetic since eight years receiving oral hypoglycemic agents, which were changed to insulin before operation. During his post operative course he had prolong ileus and abdomen was re-explored on the postoperative day 4. Ileal resection was carried out with ileostomy and mucus fistulae. He had persistent ileus and fever following re-exploration. Swab from surgical wound grew *E. coli* (ESBL strain), which was treated with appropriate antibiotics according to microbiological reports. Infectious disease consultant's opinion was taken on postoperative day 9, for persistent fever and intraabdominal infection. On examination surgical site showed black margins with surrounding erythematous area and skin wall edema. Patient was febrile with tachycardia, toxic look and requiring ventilatory support. His blood investigations showed polymorphonuclear leukocytosis (WBC counts 18850/cmm, 88% Polymorphs, 08% Lymphocytes, 02% eosinophil and 02% monocytes, platelet counts 2,62,000/cmm), while his liver functions and renal functions including electrolytes were normal and diabetes was controlled with insulin administration. His HbA1c on admission was 5.4. No past history of prolong immunosuppressive treatment and he was HIV nonreactive. Patient was advised third surgery. Intraoperative findings revealed extensive involvement of rectus sheath, cecum, ascending colon as shown in [Figure 1]. A skin biopsy was performed and tissue culture was sent. The complete resected part of colon was sent for histopathologic examination and tissue culture. The biopsy as well as tissue culture revealed extensive involvement of abdominal wall around surgical site and that of colon with mucormycosis [Figure 2]. Patient was treated with right hemicolectomy and resection of involved anterior abdominal wall along with amphotericin B deoxycholate infusion 1mg/kg/day and Meropenem 1gm q8h. Patient also received supportive care and antibiotic dosage were adjusted during the illness course according to creatinine clearance. Patient died on 8^th^ day after last surgery with ongoing sepsis following intra-abdominal infection.

## DISCUSSION

The most common manifestation of mucormycosis is rhino-cerebral form.[5] Most affected patients are diabetics, especially poorly controlled with ketoacidosis, patients on immunosuppressive treatment, desferioxime treatment and HIV and patients with malignancies.[6] Skin and soft tissue nosocomial infection has been described in hospital settings with mucormycosis.[7] These outbreaks/ sporadic cases have been linked with contaminated bandages, adhesive tapes,[8] needles, and tongue depressors used to construct splints for intravenous and arterial cannulation sites.[9,10]

Intact mucosal and endothelial barriers serve as structural defense mechanism and prevent tissue invasion and angioinvasion by Zygomycetes. Primary cutaneous zygomycosis is seen in relation to disruption of skin integrity mainly in immunocompromised patients, patients who have burns or severe soft tissue trauma (road traffic accident), and very premature neonates; it has been reported rarely in patients who have apparently normal skin.[11] Cutaneous zygomycosis typically starts as erythema and induration of the skin at a puncture site and progresses to necrosis. Extension to the subcutaneous tissue or bone is common in patients who have delayed or ineffectively treated cutaneous zygomycosis. Necrotizing fasciitis has been reported in cases of cutaneous zygomycosis and carries an extremely poor prognosis.

Gastrointestinal involvement with zygomycosis is rare and usually presents as necrotizing enterocolitis in premature neonates and a mass like appendiceal or ileal lesion in neutropenic patients.[1,12] Peritonitis due to mucormycosis has been rarely described in patients undergoing CAPD.[13–15] These infections are very rare. Diagnosis of gastrointestinal zygomycosis usually is delayed, because the nonspecific presentation requires a high degree of suspicion.

Our case developed rare gastrointestinal involvement from abdominal wall infection which is not so rare for following possible reasons in addition to immunocompromised state:

1. Mucormycosis was not suspected and diagnosed with second surgery and hence patient has not received appropriate surgical debridement and amphotericin B therapy,
2. Swab culture from surgical site is insufficient diagnostic tool for mucormycosis which obviously grew only *E. coli* only. We should bear in mind that the possibility of mycotic infection including mucormycosis in abdominal wall infection after operation in immunocomprimised state and start appropriate antifungal therapy as soon as possible.

Diagnosis can be made through visualization of broad, aseptate, 90° branching, ribbon like mucor hyphae in skin biopsy histopathological examination and culture in Sabouraud dextrose agar.[6] The disease site and host factors are key determinants of prognosis for zygomycosis. Successful treatment of zygomycosis largely depends on early diagnosis, correction or reversal of the underlying predisposing factors, adequate surgical resection/ debridement of infected tissue, and rapid initiation of effective systemic antifungal therapy in form of Amphotericin B.[6,16]

## CONCLUSION

In suspected surgical site fungal infection, skin biopsy from surgical wound along with culture is an important part of diagnostic work up. Surgeons should not solely rely on swab culture from surgical wound for diagnosis of surgical site infection, especially when edges of wound showed areas of necrosis with/ without vasculitis. Early diagnosis and prompt surgical debridement along with appropriate antifungal treatment can improve prognosis of patient with surgical site mucormycosis.

## References

[1] Ribes J, Vanover-Sams C, Baker D (2000). Zygomycetes in human disease. Clin Microbiol Rev.

[2] Sugar AM, Mandell GL, Bennett JE, Dolin R (2000). Agents of mucormycosis and related species. Mandell, Douglas, and Bennett's principles and practice of infectious diseases.

[3] Yeung CK, Cheng VC, Lie AK, Yuen KY (2001). Invasive disease due to *Mucorales* a case report and review of the literature. Hong Kong Med J.

[4] Chakrabarti A, Das A, Sharma A, Panda N, Das S, Gupta KL (2001). Ten years' experience in zygomycosis at a tertiary care centre in India. J Infect.

[5] Roden MM, Zaoutis TE, Buchanan WL, Knudsen TA, Sarkisova TA, Schaufele RL (2005). Epidemiology and outcome of zygomycosis: a review of 929 reported cases. Clin Infect Dis.

[6] Kontoyiannis DP, Lewis RE (2006). Invasive zygomycosis: update on pathogenesis, clinical manifestations, and management. Infect Dis Clin North Am.

[7] Robledo-Ogazón F, Lizaola-Pérez B, Mier-Giraud F, Bojalil-Durán L (2007). Abdominal wall infection due to Mucormycosis. Case report. Cir Cir.

[8] Mead JH, Lupton GP, Dillavou CL, Odom RB (1979). Cutaneous Rhizopus infection. Occurrence as a postoperative complication associated with an elasticized adhesive dressing. JAMA.

[9] Mitchell SJ, Gray J, Morgan ME, Hocking MD, Durbin GM (1996). Nosocomial infection with Rhizopus icrosporus in preterm infants: association with wooden tongue depressors. Lancet.

[10] Baraia J, Muñoz P, Bernaldo de Quirós JC, Bouza E (1995). Cutaneous mucormycosis in a heart transplant patient associated with a peripheral catheter. Eur J Clin Microbiol Infect Dis.

[11] Andresen D, Donaldson A, Choo L, Knox A, Klaassen M, Ursic C (2005). Multifocal cutaneous mucormycosis complicating polymicrobial wound infections in a tsunami survivor from Sri Lanka. Lancet.

[12] Park YS, Lee JD, Kim TH, Joo YH, Lee JH, Lee TS (2002). Gastric mucormycosis. Gastrointest Endosc.

[13] Branton MH, Johnson SC, Brooke JD, Hasbargen JA (1991). Peritonitis due to Rhizopus in a patient undergoing continuous ambulatory peritoneal dialysis. Rev Infect Dis.

[14] Nannini EC, Paphitou NI, Ostrosky-Zeichner L (2003). Peritonitis due to Aspergillus and zygomycetes in patients undergoing peritoneal dialysis: report of 2 cases and review of the literature. Diagn Microbiol Infect Dis.

[15] Fergie JE, Fitzwater DS, Einstein P, Leggiadro RJ (1992). Mucor peritonitis associated with acute peritoneal dialysis. Pediatr Infect Dis J.

[16] Jiménez C, Lumbreras C, Aguado JM, Loinaz C, Paseiro G, Andrés A, Morales JM, Sánchez G, García I, del Palacio A, Moreno E (2002). Successful treatment of mucor infection after liver or pancreas-kidney transplantation. Transplantation.

